# Identification of membrane proteins regulated by ADAM15 by SUSPECS proteomics

**DOI:** 10.3389/fmolb.2023.1162504

**Published:** 2023-06-14

**Authors:** Matteo Calligaris, Chun Y. Yang, Simone Bonelli, Donatella Pia Spanò, Stephan A. Müller, Stefan F. Lichtenthaler, Linda Troeberg, Simone D. Scilabra

**Affiliations:** ^1^ Proteomics Group of Fondazione Ri.MED, Research Department IRCCS ISMETT (Istituto Mediterraneo per i Trapianti e Terapie ad Alta Specializzazione), Palermo, Italy; ^2^ Department of Pharmacy, University of Pisa, Pisa, Italy; ^3^ Centre for OA Pathogenesis Versus Arthritis, Kennedy Institute of Rheumatology, University of Oxford, Oxford, United Kingdom; ^4^ STEBICEF (Dipartimento di Scienze e Tecnologie Biologiche Chimiche e Farmaceutiche), Università degli Studi di Palermo, Palermo, Italy; ^5^ German Center for Neurodegenerative Diseases (DZNE), Munich, Germany; ^6^ Neuroproteomics, School of Medicine, Klinikum Rechts der Isar, Technical University of Munich, Munich, Germany; ^7^ Munich Cluster for Systems Neurology (SyNergy), Munich, Germany; ^8^ Norwich Medical School, Bob Champion Research and Education Building, University of East Anglia, Norwich, United Kingdom

**Keywords:** ADAMs, ADAM15, metalloproteases, chondrocytes, osteoarthritis, SPECS, proteomics, mass spectrometry-LC-MS/MS

## Abstract

ADAM15 is a member of the disintegrin-metalloproteinase family of sheddases, which plays a role in several biological processes including cartilage homeostasis. In contrast with well-characterized ADAMs, such as the canonical sheddases ADAM17 and ADAM10, little is known about substrates of ADAM15 or how the enzyme exerts its biological functions. Herein, we used “surface-spanning enrichment with click-sugars (SUSPECS)” proteomics to identify ADAM15 substrates and/or proteins regulated by the proteinase at the cell surface of chondrocyte-like cells. Silencing of ADAM15 by siRNAs significantly altered membrane levels of 13 proteins, all previously not known to be regulated by ADAM15. We used orthogonal techniques to validate ADAM15 effects on 3 of these proteins which have known roles in cartilage homeostasis. This confirmed that ADAM15-silencing increased cell surface levels of the programmed cell death 1 ligand 2 (PDCD1LG2) and reduced cell surface levels of vasorin and the sulfate transporter SLC26A2 through an unknown post-translational mechanism. The increase of PDCD1LG2 by ADAM15 knockdown, a single-pass type I transmembrane protein, suggested it could be a proteinase substrate. However, shed PDCD1LG2 could not be detected even by a data-independent acquisition mass spectrometry, a highly sensitive method for identification and quantification of proteins in complex protein samples, suggesting that ADAM15 regulates PDCD1LG2 membrane levels by a mechanism different from ectodomain shedding.

## 1 Introduction

A disintegrin and metalloproteinase 15 (ADAM15) is a member of the ADAM family of membrane-tethered metalloproteinases, which are involved in the proteolytic release of transmembrane proteins, in a process termed “ectodomain shedding”. ADAMs have been reported to shed growth factors, cytokines, receptors and adhesion molecules, thereby playing important roles in a wide variety of biological processes (Dreymueller et al., 2015; Hsia et al., 2019). Unlike other members of the family, such as ADAM10 and ADAM17, whose substrate repertoire and pathophysiological functions have been extensively characterized, understanding of ADAM15 activity and mechanism of action is still in its infancy. ADAM15 has been shown to be an active protease *in vitro* (Maretzky et al., 2009), but no substrates of ADAM15 have been validated *in vivo* (Maretzky et al., 2009). Loss of ADAM15 in mice leads to a number of phenotypic abnormalities and altered physio-pathological processes, including enhanced tissue remodeling and reduced neovascularization (Horiuchi et al., 2003; Jana et al., 2020; Chute et al., 2022). Furthermore, ADAM15 has been shown to be chondroprotective in mice (Bohm et al., 2005). Previous reports showed that its expression was increased in human osteoarthritic chondrocytes and that its deficiency accelerated spontaneous development of osteoarthritis (OA) upon ageing (Bohm et al., 1999; Bohm et al., 2005). Furthermore, stable expression of ADAM15 enhanced adhesion of chondrocyte-like cells to type II and VI collagen and reduced their apoptosis (Bohm et al., 2005; Bohm et al., 2010; Fried et al., 2012). Despite its clear role in regulating cartilage homeostasis, little is known about how ADAM15 exerts its biological functions. A systemic secretome analysis using state-of-the-art mass spectrometry-based methods failed to identify ADAM15 substrates in chondrocytes and chondrocyte-like cells, arguing against the assumption that ADAM15 exerts its biological functions by releasing transmembrane proteins (Yang et al., 2020; Yang et al., 2022). Additionally, in agreement with these studies, ADAM15 has been shown to regulate pathological neovascularization in the retina independently of its catalytic activity, indicating that ADAM15 has at least some functions that do not require its proteolytic potential (Maretzky et al., 2014). Since ADAM15 is transported to the cell surface after furin cleavage of its pro-domain, we consider the plasma membrane to be the main location where ADAM15 is likely to exert its functions, either by shedding its substrates or by interacting with other membrane proteins. Thus, in order to identify proteins regulated by ADAM15 we used an innovative proteomic workflow, namely “surface-spanning protein enrichment with click sugars” (SUSPECS) (Herber et al., 2018), to analyse levels of cell membrane proteins in ADAM15-silenced chondrocyte-like HTB94 cells. This workflow enabled detection of over twice as many transmembrane proteins regulated by ADAM15 as a standard proteomic approach, and enabled us to identify 13 novel proteins regulated by ADAM15, including vasorin (VASN), the sulfate transporter (SLC26A2) and programmed cell death 1 ligand 2 (PDCD1LG2).

## 2 Materials and methods

### 2.1 Sample preparation for standard proteomics

HTB94 human chondrosarcoma cell line was a kind gift from Prof Hideaki Nagase and previously obtained from American Culture Type Collection (Manassas, VA). HTB94 were seeded in 6-well plates and grown to confluence. Cells were then collected in STET lysis buffer (150 mM NaCl, 50 mM Tris, pH 7.5, 2 mM EDTA, 1% Triton-X-100). 25 μg of proteins were subjected to filtered-aided sample preparation (FASP) with 10 kDa Vivacon spin filters (Sartorius, Germany) (Wisniewski et al., 2009). In brief, proteins were sequentially digested with 0.5 μg LysC (Promega, Madison, United States) overnight and 0.5 μg trypsin (Promega, Madison, United States) for 4 h at 37°C. Peptides were desalted by using stop-and-go extraction (STAGE) Tips on reverse phase C18 (Supelco Analytical Products, part of Sigma-Aldrich, Bellefonte, United States), and eluted in 40 μL of 60% acetonitrile in 0.1% formic acid (Rappsilber et al., 2003). The volume was reduced in a SpeedVac (Thermo Fisher Scientific, Waltham, United States) and the peptides resuspended in 0.1% formic acid for LC-MS/MS analysis. Approximately 1 μg of peptides were injected for LC-MS/MS analysis.

### 2.2 Sample preparation for SUSPECS analysis

#### 2.2.1 Metabolically labeling of HTB94 cells

HTB94 cells were plated in a 6-well plate until 70% confluent, and then transiently transfected with an ADAM15 siRNA or non-targeting control siRNA (s16681 or 4390844, respectively; Thermo Fisher Scientific) by using jetPRIME transfection reagent (Polyplus, Illkirch, France). Cells were then cultured at 37°C for 12 h, before addition of 50 μM ManNAz sugar (Thermo Fisher Scientific) to the culture for another 24 h. Cells were washed twice with 2 mL ice cold PBS (pH 7.4), before adding 25 μM sulfo-DBCO-biotin conjugate in ice cold PBS, and incubating for 2 h at 4°C in the dark. Cells were washed twice with 10 mL ice cold PBS to remove excess sulfo-DBCO-biotin conjugate, and lysed in 2 mL STET lysis buffer. Cell lysates were centrifuged (5 min, 4,000 × g, 4°C) and the resultant supernatants filtered through a 0.2 mm Corning syringe filter and collected in a 15 mL tube. Biotinylated proteins were pulled down using streptavidin.

#### 2.2.2 Streptavidin-agarose pulldown of “clicked” glycoproteins

300 μL of Pierce high capacity streptavidin-agarose (Thermo Fisher Scientific) was loaded to a Poly-Prep chromatography column (Bio-Rad Laboratories, Hercules, United States) and “clicked” cell lysates were loaded onto the column. After washing with 10 mL wash buffer (50 mM Tris-HCl, pH 7.5, 150 mM NaCl, 2 mM EDTA, 1% Triton) to remove non-specifically bound material, streptavidin-agarose beads were re-suspended in 500 μL wash buffer and transferred to new Eppendorf tubes. Beads were centrifuged (1 min, 7,000 × g, room temperature) and peptides eluted using elution buffer (62.5 mM Tris. HCl, pH 6.8, 8 M urea, 2% SDS, 10% glycerol, 5% mercaptoethanol, 0.002% bromphenol-blue) with 3 mM biotin, and boiled (95°C, 5 min). Eluates were loaded onto a 10% acrylamide gel for SDS-PAGE electrophoresis. The gel was stained using Coomassie brillant blue (30–60 min), and de-stained overnight with 10% acetic acid. Qualitatively equal gel slices were then excised from the gel and proteins in the gel slices digested with trypsin using the in-gel digestion protocol.

#### 2.2.3 In-gel digestion

In-gel digestion was performed as previously described (Shevchenko et al., 2006). In brief, protein lanes from the de-stained acrylamide gel were sliced out vertically and divided into 8 equal horizontal pieces above the BSA band (the thickest band at around 64 kDa on the gel) and 6 equal horizontal pieces below the BSA band. Pieces were further sliced into 1 mm^3^ cubes, collected in a 96-well plate and incubated in 150 μL of acetonitrile (ANC) (room temperature, 10 min). Gels were de-hydrated using vacuum centrifugation, then 50 μL of 10 mM dithiothreitol (DTT) solution in 100 mM ammonium bicarbonate (ABC) was added to each well (55°C, 30 min). 55 mM iodoacetamide (IAA) in 100 mM ABC was added to the gel pieces, and incubated for 20 min. Gels were then de-hydrated using 150 μL ANC. 50 μL of 3 ng/μL trypsin in 10 mM ABC; 10% ACN was added to cover each gel piece completely, and trypsin incubation performed on ice for 30 min. ABC was then added to cover the gel pieces completely, the 96-well plate covered with its lid, and placed into a 37°C incubator overnight. On the following day, 5% formic acid (FA) and ACN were mixed (1:2 volume: volume ratio) immediately before use and 100 μL of the mixture added to wells before incubation at room temperature with gentle shaking for 15 min to extract fragmented peptides. The resultant supernatant was collected in an Eppendorf tube, and the extraction step repeated (generally 3 times) until gel pieces were completely de-hydrated. Peptides in the supernatant were de-hydrated using vacuum centrifugation and reconstituted in 33 μL 0.1% FA.

### 2.3 Proteomic analysis using LC-MS/MS

To perform liquid chromatography tandem mass spectrometry (LC-MS/MS), approximately 1 μg of peptides from each sample were loaded onto the nanoLC system (EASY-nLC 1,000, Proxeon) using an in-house packed C18 column (50 cm × 75 μm ID, ReproSil-Pur 120 C18-AQ, 1.9 μm, Dr. Maisch GmbH, Germany) with a binary gradient (Table 2)12 of water and acetonitrile containing 0.1% FA at a column temperature of 50 °C. The nanoLC system was coupled with a Q-Exactive mass spectrometer (Thermo Fisher Scientific) via an nanospray ex ion source system (Proxeon) online. Mass spectra were acquired at a resolution of 70,000 and the top 10 peptide ions exceeding an intensity of 2.5 × 10^4^ were chosen for collision-induced dissociation to sequence peptides via fragmentation. The resultant fragment ion spectra were acquired at a resolution of 17,500. A dynamic exclusion of 120 s was used for peptide fragmentation.

### 2.4 Analysis of the proteomic data

Data generated by LC-MS/MS was analysed using Maxquant 1.5.2.8. (Max-Planck Institute Munich, Germany). Proteomic data generated were compared against a reviewed canonical FASTA database of *Homo sapiens* from UniProt. Two missed cleavages were allowed when performing database searches. False discovery rates (FDR) of both peptides and proteins were adjusted to less than 5% using a decoy proteomic data base using the same criteria as the real canonical FASTA database. Label-free quantification (LFQ) for protein abundance was only performed when at least two ratio counts of unique peptides were detected in each experimental conditions (non-targeting vs. knockdown). Additionally, only unique peptides were used for LFQ. The LFQ values were transformed to log2-based values, tested for normal distribution by the Kolmogorov–Smirnov test and a two-sided heteroscedastic student’s t-test was used to perform statistical analysis of significance. A *p*-value less than 5%, corrected with the FDR, was set as the significance threshold.

### 2.5 Validation of SUSPECS proteomics hits

HTB94 cells were seeded in 6-well plates and ADAM15 knocked down using siRNA and jetPRIME transfection reagent as described above. Total RNA was extracted from cultured cells using an RNeasy Mini Kit according to the manufacturer’s guide (Qiagen, Hilden, Germany). The concentration of purified RNA was determined using a Nanodrop 1,000 (Thermo Fisher Scientific). Then, RNA (300–600 ng) was reversely transcribed to cDNA using the High-Capacity cDNA Reverse Transcription Kit (Thermo Fisher Scientific) according to the manufacturer’s guide. qPCR was performed using TaqMan Fast universal PCR Master Mix (Thermo Fisher Scientific). Master Mix (6 μL per reaction) was mixed with nuclease-free water (0.4 μL per reaction) and a specific primer for the gene of interest (0.6 μL per reaction; ADAM15—No. Hs00187052_m1; RPLP0—No.Hs99999902_m1; VASN—Hs01392409_m1; PDCD1LG2—Hs00228839_m1 and SLC26A2—Hs00164423_m1; TaqMan Gene Expression Assays, Applied Biosystems, part of Thermo Fisher Scientific). cDNA was diluted 5-fold and added to the qPCR reagent mixture. qPCR was performed using a Rotor-Gene 6,000 Real Time PCR machine (Corbett Research). ∆Ct was calculated by subtracting the Ct value of the gene of interest from the Ct value of the housekeeping gene (*RPLP0*), and fold change (FC) was calculated using the formula FC = 2-(∆∆Ct).

For immunoblot analysis, HTB94 cells treated with ADAM15 or control siRNA were collected in STET lysis buffer (50 mM Tris, pH 7,5, 150 mM NaCl, 2 mM EDTA, 1% Triton) containing protease inhibitor cocktail (1:100, Sigma Aldrich). Protein concentration was measured using a colorimetric 660 nm microBCA assay (Thermo Fisher Scientific) and 20 μg of protein loaded onto an acrylamide gel for SDS-PAGE. Gels were blotted to a PVDF membrane for detection with the following antibodies: anti-ADAM15 (clone EPR5619, cat. ab124698), anti-VASN (clone EPR10901) and anti-PDCD1LG2 (clone EPR1163) all from Abcam (Cambridge, United Kingdom), and SLC26A2 (HPA058090, Atlas Antibodies AB, Bromma, Sweden), and CANX (ADI-SPA-860-F, Enzo Life Science, Lausen, Switzerland). Bands corresponding to each protein were quantified using Image Lab software (Bio-Rad, Hercules, CA, United States) and a two-sided Student’s t-test was used to evaluate proteins statistical significance. A *p*-value less than 0.05 was set as the significance threshold.

### 2.6 Data-independent acquisition mass spectrometry

#### 2.6.1 Sample preparation

HTB94 were grown in a six-well plate and treated with ADAM15 or non-targeting siRNA for 48 h. Then, cells were incubated with 2 mL serum-free media for 24 h. Conditioned media were collected and applied to filter aided sample processing (FASP) (Wisniewski et al., 2009). Briefly, proteins were reduced by the addition of 1 M dithiothreitol (DTT, Thermo Fischer Scientific) in 100 mM Tris/HCl, 8 M urea pH 8.5 for 30 min at 37°C. Proteins were then alkylated in 50 mM iodoacetamide (IAA, Thermo Fischer Scientific) for 5 min at room temperature and washed twice in 100 mM Tris/HCl, 8 M urea pH 8.0 at 14,000× g for 30 min. Proteins were digested with 0.2 μg LysC (Promega, Madison, United States) in 25 mM Tris/HCl, 2 M urea pH 8.0 overnight and with 0.1 μg trypsin (Promega) in 50 mM ammonium bicarbonate for 4 h. Resulting peptides were desalted by stop-and-go extraction (STAGE) on reverse phase C18 (Supelco Analytical Products, part of Sigma-Aldrich, Bellefonte, United States), and eluted in 40 μL of 60% acetonitrile in 0.1% formic acid (Rappsilber et al., 2003). Then, the volume was reduced in a SpeedVac (Thermo Fisher Scientific) and the peptides resuspended in 20 μL of 0.1% formic acid. Peptide concentration was measured by a NanoDrop microvolume spectrophotometer (Thermo Fischer Scientific) and 1 μg of peptides were applied to LC-MS/MS analysis.

#### 2.6.2 LC-MS/MS

To achieve high sensitivity, a nanoLC system (Vanquish Neo UHPLC—part of Thermo Scientific) using an Acclaim PEPMap C18 column (25 cm × 75 µm ID, Thermo Scientific, Waltham) was coupled online to an Exploris 480 mass spectrometer (Thermo Fischer Scientific). Peptides were separated using a 130 min binary gradient of water and acetonitrile containing 0.1% formic acid. Data-independent acquisition (DIA) was performed using a MS1 full scan followed by 60 sequential DIA windows with an overlap of 1m/z and window placement optimization option enabled. Full scans were acquired with 120,000 resolution, automatic gain control (AGC) of 3 × 10^6^, and maximum injection time of 50 ms. Afterwards, 60 isolation windows were scanned with a resolution of 30,000, an AGC of 8 × 10^5^ and maximum injection time was set as auto to achieve the optimal cycle time. Collision induced dissociation fragmentation was induced with 30% of the normalized HCD collision energy. The data were analysed by the software DIA-NN (version 1.8.1) by using a predicted library generated from *in silico* digested human Uniprot reference database involving cuts at K* and R*, two missed cleavage allowed, minimal peptide length set a 6, and which consisted of 20,923 proteins, 31,382 protein groups, 6894911 precursors in 2140232 elution groups. The false discovery rate for peptide and protein identification was set at 0.01%. Label-free quantification (LFQ) was used for protein quantification. The LFQ values were log2 transformed and a two-sided Student’s t-test was used to evaluate proteins significantly regulated between ADAM15 KD and controls (*n* = 3). A *p*-value less than 0.05 was set as the significance threshold.

## 3 Results

### 3.1 Identification of cell membrane proteins regulated by ADAM15 in HTB94 chondrosarcoma-like cells by SUSPECS

**FIGURE 1:**
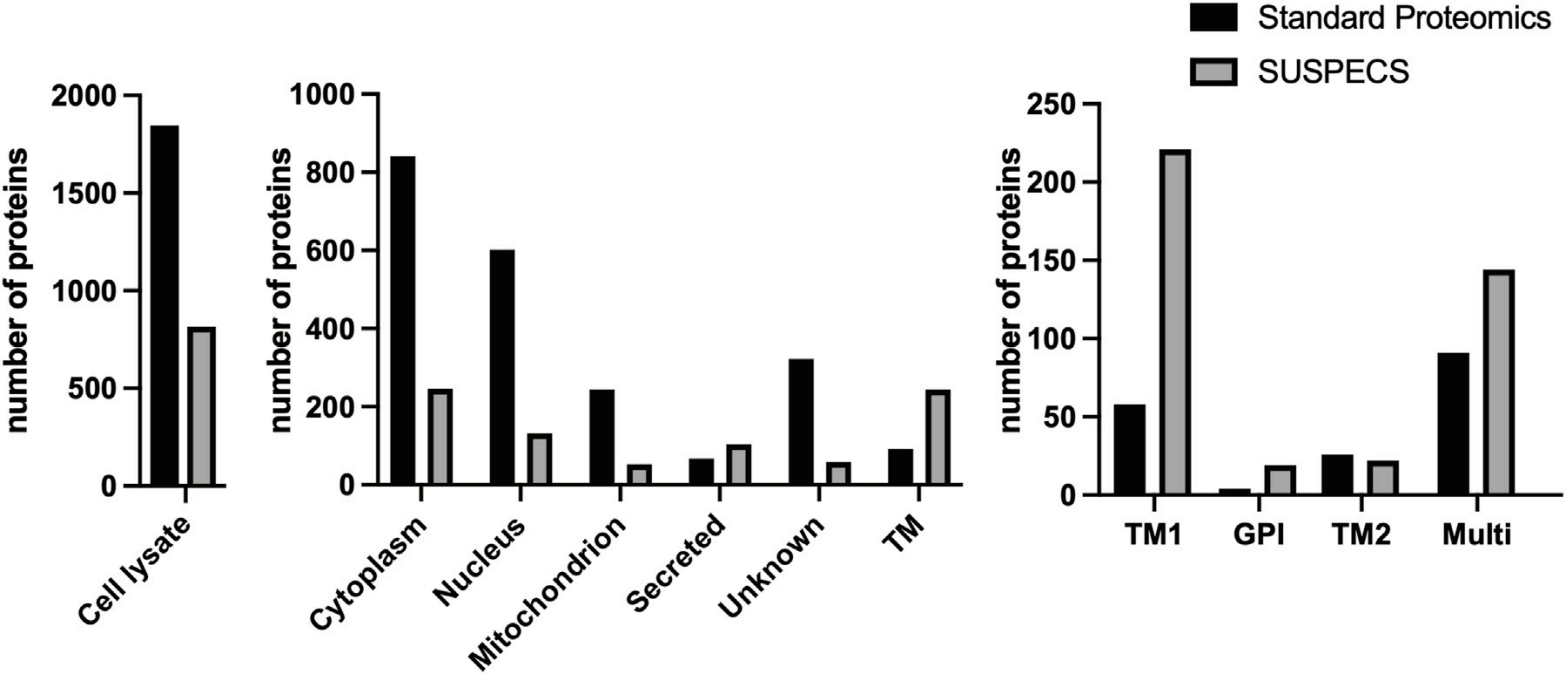
Number and subcellular location of proteins detected by standard proteomics and SUSPECS. 1848 proteins were identified by standard proteomics in cell lysates of ADAM15 KD and control HTB94 cells, including 841 cytoplasmic, 602 nuclear, 244 mitochondrial, 67 secreted and 322 proteins whose subcellular location is unknown. Moreover, 92 transmembrane proteins (TM) were detected by the analysis, of which 58 were type I (TM1), 4 GPI-anchored, 26 type II (TM2) and 91 multipass proteins. SUSPECS identified 816 proteins, including 246 cytoplasmic, 132 nuclear, 53 mitochondrial, 67 secreted, 59 proteins whose subcellular location is unknown, and 244 transmembrane proteins (TM). Among these, 221 were type I, 19 GPI-anchored, 22 type II and 144 multipass transmembrane proteins, based on annotations in the reviewed canonical FASTA database of Homo sapiens from UniProt.

We silenced expression of ADAM15 in chondrocyte-like HTB94 cells using siRNA and analysed cell lysates by label-free quantitative proteomics (Wisniewski et al., 2009) in order to identify proteins whose cellular abundance was regulated by the proteinase. A total of 1849 proteins were identified (Figure 1; Supplementary Table S1), but only 92 (approximately 5%) of these were transmembrane proteins according to their Uniprot annotations (Figure 1). The majority of proteins detected by this method were cytoplasmic (841 proteins) and nuclear (602 proteins). Among the transmembrane proteins, 58 were type I (which the vast majority of ADAM substrates fall into (Edwards et al., 2008; Hsia et al., 2019)), 26 were type II, 4 were GPI and 8 were type III/IV transmembrane proteins (Figure 1).

**FIGURE 2:**
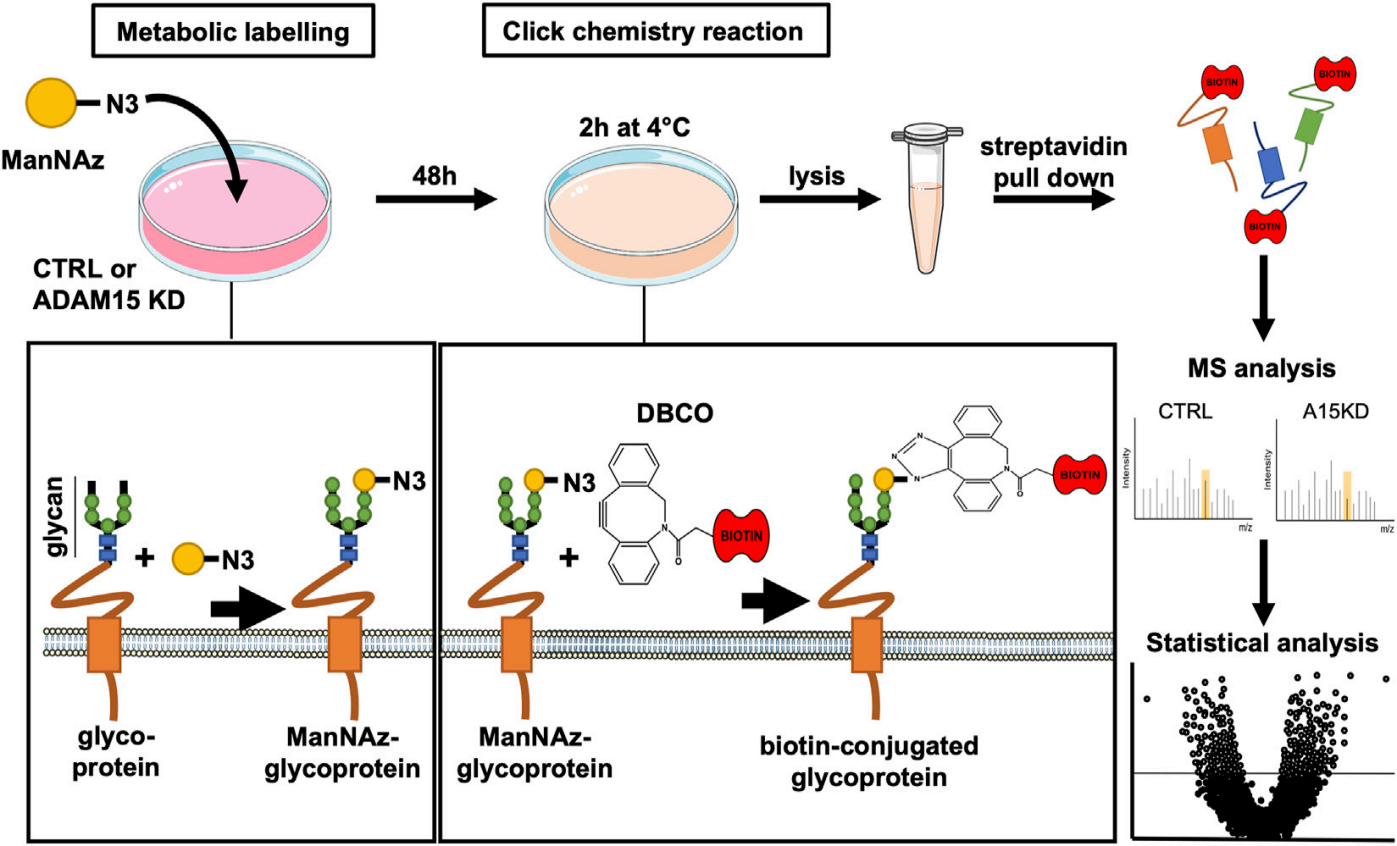
Schematic illustration of SUSPECS. To pull-down newly synthesised cell membrane glycoproteins whose abundance could be affected by changes in ADAM15 expression, tetra-acetylated N-azidoacetylmannosamine (ManNAz) was added to the culture medium for metabolic labeling of HTB94 cells after ADAM15 expression was knocked down by a specific siRNA (ADAM15 KD) or treated with non-targeting control siRNA (CTRL). ManNAz is permeable to the plasma membrane and is incorporated as modified terminal N-azido-sialic acid into the glycan moiety of glycoproteins during their synthesis. Glycoproteins containing ManNAz are “clicked” using sulfo-dibenzylcyclooctyne-biotin conjugate (DBCO). The DBCO moiety forms a covalent bond with the azide group of the azido-sialic acids N3 group of glycoproteins. Since the sulfo-DBCO-biotin is membrane impermeable, sulfo-DBCO-biotin will only “click” glycoproteins on the plasma membrane, which can then be pulled down using streptavidin-agarose. Enriched glycoproteins are separated via SDS-PAGE electrophoresis. After fractionation, proteins are in-gel digested and analysed by mass spectrometry using label-free quantification. Finally, protein changes are represented with a volcano plot, which combines a measure of statistical significance (e.g., a *p*-value from a Student’s t-test) with the magnitude changes.

Given the restricted number of transmembrane proteins detected by the initial analysis, we chose to apply the SUSPECS workflow to enrich membrane proteins from HTB94 cells. SUSPECS enrichment of cell membrane proteins was developed to reduce sample complexity of cell lysates and allow mass-spectrometry detection of low abundance membrane proteins whose signal could otherwise be obscured by highly abundant intracellular proteins (Herber et al., 2018). SUSPECS involves metabolically labelling and click chemistry biotinylation of cell surface glycoproteins, which are then enriched by streptavidin pulldown and analysed by mass-spectrometry and LFQ (Figure 2). By using this workflow, we increased by 2.65-fold the number of transmembrane proteins identified (244 proteins, Figure 1; Supplementary Table S1). Among the transmembrane proteins identified, 211 were type-I, 22 type II and 19 were GPI-anchored proteins (Figure 1; Supplementary Table S1). As expected, the number of intracellular cytoplasmic and nuclear proteins was drastically reduced to 246 and 132 proteins, respectively (Figure 1; Supplementary Table S1).

**FIGURE 3:**
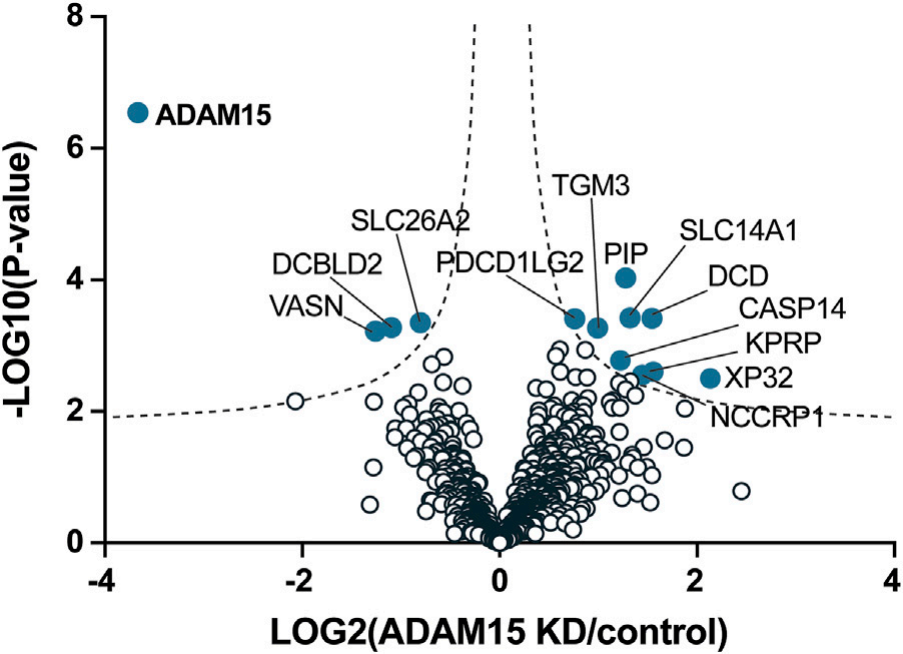
Identification of cell membrane proteins regulated by ADAM15 in HTB94 cells by SUSPECS. Data obtained from SUSPECS analysis, showing the 841 detected proteins, with log2 of the ratio between protein abundance in the surfaceome of cells transfected with siADAM15 and that of cells transfected with non-targeting control siRNA (ADAM15 KD/control) versus the log_10_ of the *p*-value. Proteins above the FDR (black dashed lines) reached the threshold of statistical significance. 13 proteins showed significant changes (solid blue circles, *n* = 6).

These data clearly showed that SUSPECS was preferential to standard proteomic analysis of cell lysates to evaluate levels of transmembrane proteins. Thus, we applied the SUSPECS workflow to HTB94 cells treated with control and ADAM15 siRNA. 13 proteins showed significant changes in abundance in the surfaceome of ADAM15-knockdown cells (depicted above the two black dashed lines as solid blue circles on Figure 3; Supplementary Table S2). Notably, ADAM15 was identified as the most significantly decreased protein with the highest magnitude of reduction in the surfaceome, indicating successful ADAM15 knockdown (Figure 3; Table 1; Supplementary Table S2). Among proteins which showed significant changes (excluding ADAM15), only 3 were decreased in the surfaceome of ADAM15-knockdown cells. All 3 were transmembrane proteins, with sulfate transporter (SLC26A2) being a multi-pass membrane protein, and vasorin (VASN) and discoidin, CUB and LCCL domain-containing protein 2 (DCBLD2) being type-1 transmembrane proteins (Figure 3; Table 1; Supplementary Tables S1, S2). Nine proteins were more abundant on the cell surface when ADAM15 expression was silenced i.e., 5 intracellular proteins (referred to by their gene names KPRP, XP32, NCCRP1, TGM3 and CASP14), two secreted proteins (DCD and PIP) and two transmembrane proteins PDCD1LG2 (a type I protein) and SLC14A1 (a multipass membrane protein), according to Uniprot annotation (Figure 3; Table 2; Supplementary Tables S1, S2).

**TABLE 1:** Proteins significantly decreased in HTB94 cells by ADAM15 knockdown.

Protein name	Protein ID	Gene name	*p*-value	Ratio	Protein location/topology
Disintegrin and metalloproteinase domain-containing protein 15	Q13444	ADAM15	2.84E-07	0.078	Type I transmembrane
Sulfate transporter	P50443	SLC26A2	4.49E-04	0.57	Multipass transmembrane
Vasorin	Q6EMK4	VASN	5.33E-04	0.46	Type I transmembrane
discoidin, CUB and LCCL domain-containing protein 2	Q96PD2	DCBLD2	5.27E-04	0.46	Type I transmembrane

Protein name: proteins decreased in ADAM15-silenced HTB94 cells above the FDR curve (FDR *p*=0.05; s_0_ = 0.1). Protein ID: Uniprot accession number of the protein. Gene name: gene name of the protein. *p-value*: for six biological replicates. *Ratio*: mean ratio of label-free quantification intensities between ADAM15 knockdown and control HTB94 cells (*n* = 6). *Protein location/topology*: information on the location and the topology of the mature protein in the cell according to the UniProt database.

**TABLE 2:** Proteins significantly increased in HTB94 cells by ADAM15 knockdown.

Protein name	Protein ID	Gene name	*p*-value	Ratio	Protein location/topology
Prolactin-inducible protein	P12273	PIP	9.38E-05	2.42	Secreted
Urea transporter 1	Q13336	SLC14A1	3.77E-04	2.49	Multipass transmembrane
Dermcidin	P81605	DCD	3.85E-04	2.91	Secreted
Programmed cell death 1 ligand 2	Q9BQ51	PDCD1LG2	3.90E-04	1.69	Type I transmembrane
Protein-glutamine gamma-glutamyltransferase E	Q08188	TGM3	5.38E-04	1.98	Cytoplasm
Caspase-14	P31944	CASP14	1.66E-03	2.33	Cytoplasm
Keratinocyte proline-rich protein	Q5T749	KPRP	2.50E-03	2.94	Cytoplasm
F-box only protein 50	Q6ZVX7	NCCRP1	2.78E-03	2.72	Cytoplasm
Skin-specific protein 32	Q5T750	XP32	3.12E-03	4.39	Unknown

*Protein name*: proteins increased in ADAM15-silenced HTB94 cells above the FDR curve (FDR *p*=0.05; s_0_ = 0.1). *Protein ID*: UniProt accession number of the protein. *Gene name*: gene name of the protein. *p-value*: for six biological replicates. *Ratio*: mean ratio of label-free quantification intensities between ADAM15 knockdown and control HTB94 cells (*n* = 6). *Protein location/topology*: information on the location and the topology of the mature protein in the cell according to the UniProt database.

### 3.2 Validation of PDCD1LG2, SLC26A2, and VASORIN as proteins regulated by ADAM15

**FIGURE 4:**
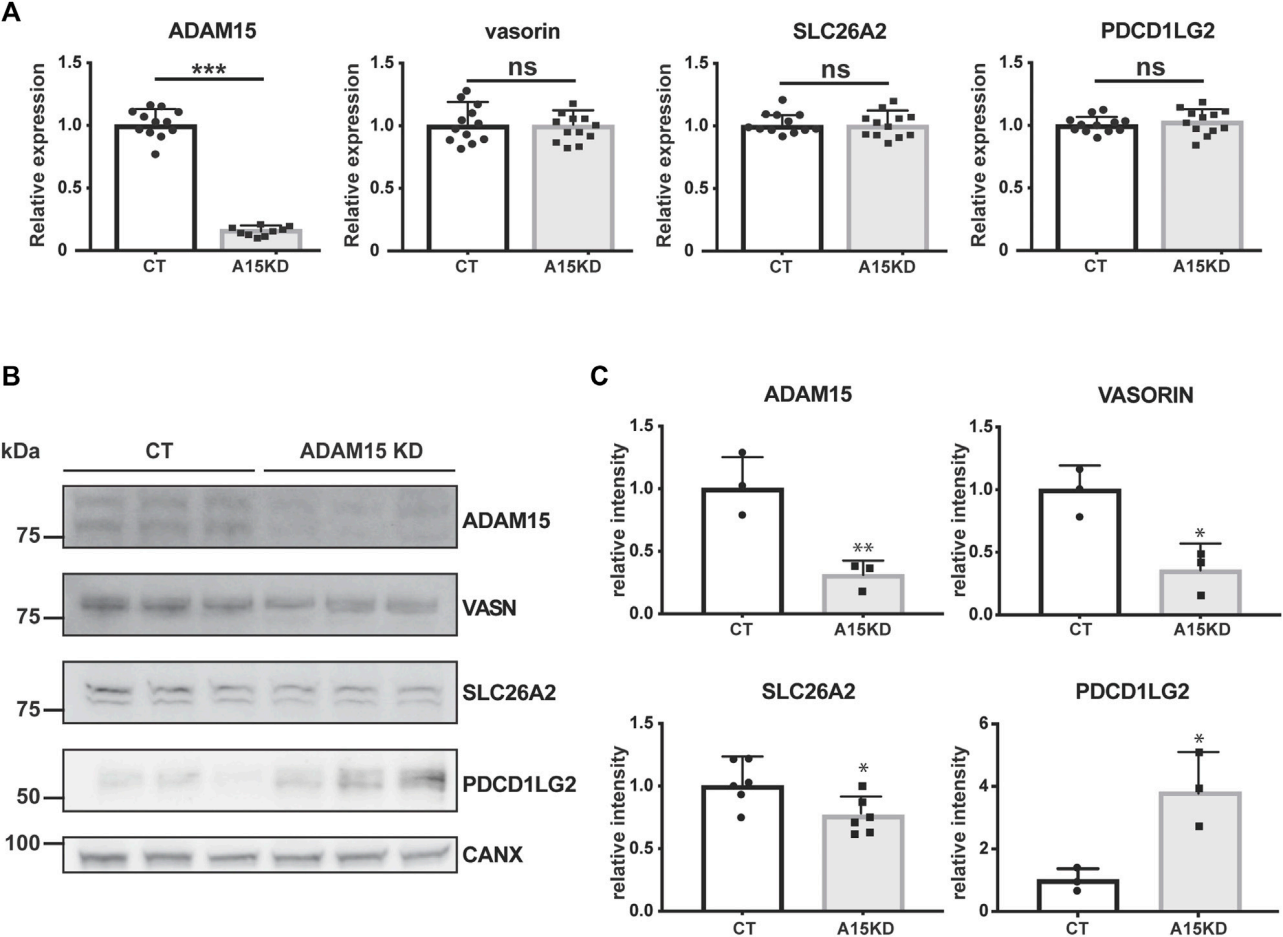
ADAM15 post-transcriptionally regulates levels of PDCD1LG2, SLC26A2, and VASN. **(A)** mRNA expression of *ADAM15*, *PDCD1LG2*, *SLC26A2,* and *VASORIN* in ADAM15 knockdown (A15KD) HTB94 cells was normalised to the housekeeping gene *RPLP0* and normalised to the average expression of the respective gene in non-targeting siRNA treated cells (CT). Statistical significance was analysed using a Student’s t-test (Data are shown as mean ± standard deviation; n. s., not significant, ***, *p* ≤ 0.001). **(B)** Immunoblots showing protein abundance of ADAM15, PDCD1LG2, SLC26A2 and VASORIN in the cell lysates of ADAM15 knockdown (ADAM15 KD) or HTB94 controls (CT). Calnexin was used as a loading control. **(C)** Densitometric quantifications of specific proteins are displayed as mean values ± standard deviation (*p < 0.05, Student’s t-test; from 3 to 6 separate experiments have been performed and analyzed).

When validating significantly altered proteins, we focused on transmembrane proteins with potential roles in cartilage homeostasis for which specific antibodies are commercially available. Thus, we analysed gene expression and protein levels for two proteins that were decreased upon ADAM15 suppression (SLC26A2 and VASN) and one protein that was augmented (PDCD1LG2) (Figure 3). SLC26A2 is a sulfate transporter and its mutation causes chondrodysplasia (Forlino et al., 2005), whilst vasorin is a TGF-β-binding protein that inhibits TGF-β signaling, which is known to have crucial roles in joint homeostasis (Ikeda et al., 2004; Shen et al., 2014). Like its homolog PD-L1, PDCD1LG2 binds to its receptor PD-1 and inhibits inflammation, particularly through inhibiting T cell activation (Latchman et al., 2001). First, we examined the effect of ADAM15 knockdown on the expression of candidates at the mRNA level. ADAM15 silencing reduced expression of the proteinase by about 80%, but had no effect on the expression of PDCD1LG2, SLC26A2 or vasorin (Figure 4A).

Next, we investigated whether ADAM15 knockdown affected abundance of these candidates at the protein level by Western blotting. In agreement with the SUSPECS proteomics results, abundance of SLC26A2 and VASN decreased in cell lysates upon ADAM15 knockdown, while levels of PDCD1LG2 increased (Figures 3B, C). These data thus confirm that ADAM15 post-transcriptionally regulated levels of SLC26A2, VASN and PDCD1LG2, three proteins with reported functions in joint homeostasis and inflammation.

### 3.3 Secretome analysis of ADAM15-silenced cells

Both SuSPECS and Western blot analysis identified PDCD1LG2 as a protein increasing in ADAM15-silenced HTB94 cells, and therefore as a putative ADAM15 substrate. In order to investigate whether ADAM15 could release PDCD1LG2, conditioned media of HTB94 cells treated with ADAM15 siRNA or non-targeting control siRNA were concentrated 40-fold by TCA precipitation and analysed by immunoblotting. PDCD1LG2 was not detected in the conditioned media of ADAM15-silenced or control cells (Supplementary Figure). Thus, we further investigated ADAM15-dependent shedding by using data-independent acquisition mass spectrometry (DIA-MS). DIA-MS is a recently-developed global MS-based strategy that allows a broader protein coverage than data-dependent acquisition (DDA) MS, which was previously used to investigate changes in the secretome of ADAM15-silenced cells (Yang et al., 2020; Yang et al., 2022).

**FIGURE 5:**
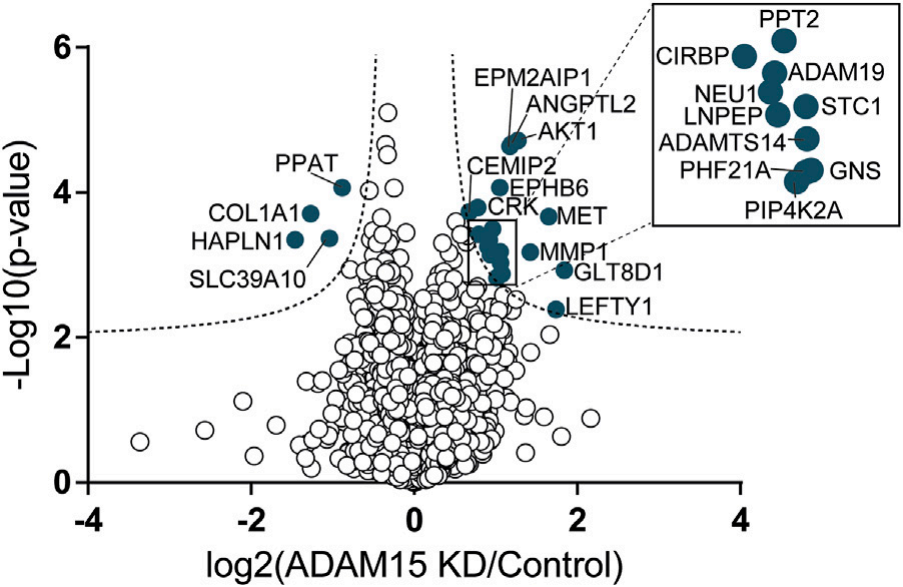
Identification of proteins regulated by ADAM15 in the secretome of HTB94 cells by DIA-MS. Data obtained from DIA-MS secretome analysis, showing the 4,378 detected proteins, with log2 of the ratio between protein abundance in the secretome of cells transfected with si*ADAM15* and that of cells transfected with non-targeting control siRNA (ADAM15 KD/control) versus the log_10_ of the *p*-value. Proteins above the FDR (black dashed lines) reached the threshold of statistical significance. 20 proteins showed significant changes (solid blue circles, *n* = 3).

DIA-MS-based secretome analysis identified and quantified levels of 4,378 proteins, but, similar to Western blotting, it did not detect PDCD1LG2 (Figure 5; Supplementary Table S3). This suggests that PDCD1LG2 was not released under the experimental conditions or, that it was released at levels too low to be detected even by a very sensitive method such as DIA-MS.

Nevertheless, the DIA-MS secretome analysis identified 24 proteins whose levels changed when ADAM15 was silenced (Figure 5; Supplementary Table S3). Four proteins were reduced, including the hyaluronan and proteoglycan link protein 1 (HAPLN1) and collagen alpha-1(I) chain (COL1A1), two proteins involved in the organization of the extracellular matrix (according to the Uniprot Gene Ontology database—GO:0030198) and associated with cartilage homeostasis and osteoarthritis (Tsezou, 2014; Jang et al., 2021). None of the reduced proteins were single-pass transmembrane proteins, and therefore canonical ADAM substrates (Supplementary Table S3). Conversely, 20 proteins were more abundant in the conditioned media of ADAM15 KD cells. Among them, the most represented group was associated with metallopeptidase activity (GO:0008237), and included ADAM19, a disintegrin and metalloproteinase with thrombospondin motifs 14 (ADAMTS-14), MMP-1 and the leucyl-cystinyl aminopeptidase (LNPEP). Two proteins with glycosidase activity, the cell surface hyaluronidase (CEMIP2) and sialidase-1 (NEU1), increased in the conditioned media of ADAM15 KD cells (Figure 5; Table 3; Supplementary Table S3) in addition to metallopeptidases, indicating that ADAM15 loss promotes the extracellular accumulation of a number of catabolic enzymes, while reducing levels of ECM structural components, such as type I collagen. Although no canonical substrates emerged from this analysis, this study identified a number of proteins at the cell surface and in the secretome that are altered when the proteinase is silenced and which are linked with cartilage homeostasis and hence potentially with the chondroprotective function of ADAM15.

**TABLE 3:** Proteins significantly increased in HTB94 cells by ADAM15 knockdown.

Protein name	Protein ID	Gene name	*p*-value	Ratio	GO biological process/Molecular function
Hyaluronan and proteoglycan link protein 1	P10915	HAPLN1	4,66E-04	0.35	extracellular matrix organization (GO:0030198)
Collagen alpha-1(I) chain	P02452	COL1A1	2,02E-04	0.41	extracellular matrix organization (GO:0030198)
Disintegrin and metalloproteinase domain-containing protein 19	Q9H013	ADAM19	4,70E-04	1.85	extracellular matrix organization (GO:0030198) metallopeptidase activity (GO:0008237)
A disintegrin and metalloproteinase with thrombospondin motifs 14	Q8WXS8	ADAMTS14	9,68E-04	2.03	extracellular matrix organization (GO:0030198)
					metallopeptidase activity (GO:0008237)
Interstitial collagenase	P03956	MMP1	6,90E-04	2.62	extracellular matrix organization (GO:0030198)
					metallopeptidase activity (GO:0008237)
Leucyl-cystinyl aminopeptidase	Q9UIQ6	LNPEP	7,50E-04	1.87	metallopeptidase activity (GO:0008237)
Cell surface hyaluronidase	Q9UHN6	CEMIP2	1,93E-04	1.56	Glycosidase (KW-0326)
Sialidase-1	Q99519	NEU1	5,78E-04	1.83	Glycosidase (KW-0326)

*Protein name*: selected proteins altered in the secretome of ADAM15-silenced HTB94 cells above the FDR curve (FDR *p*=0.05; s_0_ = 0.1). *Protein ID*: UniProt accession number of the protein. *Gene name*: gene name of the protein. *p-value*: for three biological replicates. *Ratio*: mean ratio of label-free quantification intensities between ADAM15 knockdown and control HTB94 cells (*n* = 3). *GO Biological process/Molecular function*: information on the function and biological processes in which the protein is involved according to the UniProt database.

## 4 Discussion

ADAM15 belongs to the ADAM family of membrane-tethered proteinases that mediate proteolytic release of transmembrane protein substrates into the extracellular milieu, a process known as ectodomain shedding (Lichtenthaler et al., 2018). Some members of this family, such as ADAM10 and ADAM17, have been extensively characterized. Dozens of proteins have been reported to be shed by ADAMs, pinpointing a crucial role for this family of proteinases in several biological processes, including cell-cell communication, signaling and adhesion (Edwards et al., 2008; Hsia et al., 2019). Although it has been proven to be a proteolytically active enzyme *in vitro*, the reported substrate repertoire of ADAM15 is still restricted to a few proteins, including FGFR2iiib (Maretzky et al., 2009), MICB (Duan et al., 2013), CD23 (Fourie et al., 2003), E-cadherin (Najy et al., 2008) and HB-EGF (Schafer et al., 2004), and no proteins have been validated as ADAM15 substrates *in vivo*. We previously used a number of innovative proteomic methods in an attempt to identify transmembrane proteins released by ADAM15, either in the steady-state or upon overexpressing the proteinase, but failed to identify any putative substrates ((Yang et al., 2020; Yang et al., 2022)). Similar methods to the ones we used have been effective for identifying substrates of sheddases, such as ADAM10 and BACE1 that release over 50 different proteins, and also for identifying substrates of signal peptide peptidase (SPP) sheddases that have a very limited substrate spectrum. Although physiological substrates of ADAM15 have not yet been identified, studies *in vivo* clearly show a function for ADAM15 in a number of processes, including cartilage homeostasis and tissue remodeling (Bohm et al., 2005; Jana et al., 2020; Chute et al., 2022). This suggests either that ADAM15 requires specific stimuli in order to be activated and release its target proteins, or that it can exert functions independently of its proteolytic potential. Both possibilities are supported by previous studies. For instance, ADAM15 catalytic activity was required for claudin-1-dependent regulation of growth and mobility of breast cancer cells (Mattern et al., 2019), but was not required for its role in promoting pathological neovascularization in a mouse model of oxygen-induced retinopathy (Maretzky et al., 2014). Similarly, we found that ADAM15 regulates levels of TIMP-3 in a catalytic-independent manner (Yang et al., 2022). For this reason, we here used a cutting-edge proteomic method that is specifically designed to enrich cell membrane proteins, and therefore could allow identification both of canonical ADAM15 substrates and proteins regulated by the proteinase through other catalytic-independent mechanisms.

Our analysis identified a number of transmembrane proteins that were significantly regulated by ADAM15 repression. We use immunoblotting to validate three of these proteins, SLC26A2, VASORIN, and PDCD1LG2, which may have a link with the chondroprotective function of ADAM15. Transcript levels of these proteins did not change upon ADAM15 knockdown, indicating that their regulation by ADAM15 occurs at a post-transcriptional level. SLC26A2 levels were reduced by ADAM15 knockdown. SLC26A2 is a sulfate transporter expressed in various tissues, but the only tissue affected by loss-of-function mutations of SLC26A2 are cartilage and bone (Haila et al., 2001; Forlino et al., 2005). Its downregulation or mutation results in glycosaminoglycan undersulfation in humans and mice (Haila et al., 2001; Forlino et al., 2005) and, depending on the severity of the mutation, leads to a spectrum of cartilage and skeletal abnormalities, ranging from a non-lethal recessive form of multiple epiphyseal dysplasia, to diastrophic dysplasia, postnatally lethal atelosteogenesis type 2, and embryonically lethal achondrogenesis 1B (Hastbacka et al., 1996a; Superti-Furga et al., 1996a; Hastbacka et al., 1996b; Superti-Furga et al., 1996b). The fact that SLC26A2 mutation displays dose-dependent effects on cartilage is particularly relevant because our data suggest that ADAM15 effects on SLC26A2 are mild. Reductions in the sulfate transporter function of SLC26A2 by ADAM15 are likely to reduce glycosaminoglycan sulfation. Since glycosaminoglycan sulfation is known to be critical in determining growth factor bioavailability, modulating outside-in signals through cell–cell and cell–ECM interactions, and in creating a microenvironment critical for development and homeostasis (Soares da Costa et al., 2017), slight but chronic undersulfation may have profound impacts on joint health later in life (Cortes et al., 2009; Kraushaar et al., 2013). Further studies are thus warranted to dissect the molecular mechanism by which ADAM15 supports SLC26A2 localisation.

Similar to SLC26A2, vasorin levels were reduced by ADAM15 knockdown in HTB94 cells. Vasorin directly binds to transforming growth factor-beta (TGF-β) and attenuates TGF-β signaling, which contributes to cartilage homeostasis (Shen et al., 2014; Wang et al., 2016). Indeed, the TGF-β-Smad3 pathway has been shown to have protective and catabolic effects, with mice lacking Smad3 (both whole body knockout and cartilage-specific knockout) developing accelerated OA (Yang et al., 2001; Chen et al., 2012), while inhibition of TGF-β signalling in subchondral bone mesenchymal stem cells protected mice from OA (Zhen et al., 2013). This suggests that ADAM15 may exert its chondroprotective function in part by limiting TGF-β signaling in chondrocytes.

PDCD1LG2 showed increased expression on the surface of ADAM15-knockdown cells. Like its homolog PD-L1, PDCD1LG2 binds to its receptor PD-1 and inhibits inflammation, particularly through inhibiting T cell activation (Latchman et al., 2001). Recently, PDCD1LG2 has been associated with bone loss in arthritis, in both mouse models and patients (Greisen et al., 2020). This suggests that ADAM15 may promote inflammation by downregulating abundance of PDCD1LG2 (Charrier-Hisamuddin et al., 2008). The mechanism by which ADAM15 reduces PDCD1LG2 levels is not yet clear. Its increased levels at the cell surface of ADAM15 knockdown cells suggest that PDCD1LG2 could be a substrate of the proteinase, but we were not able to detect shed PDCD1LG2 by high-resolution data-independent acquisition proteomics, and, similarly, immunoblotting failed to detect shed PDCD1LG2 in the conditioned medium of HTB94 cells (Supplementary Figure). This may be due to technical reasons (such as sensitivity of the PDCD1LG2 antibody or of the mass spectrometry-based methods), so we cannot exclude the possibility that PDCD1LG2 is indeed released by ADAM15. However, the high sensitivity of the DIA-MS approach suggests that ADAM15 regulates PDCD1LG2 surface levels by a mechanism different from shedding.

DIA mass spectrometry has been recently developed as a proteomic method that has greater sensitivity and greater dynamic range than data-dependent acquisition mass spectrometry and other methods based on chemical labelling workflows (e.g., iTRAQ and TMT) (Zhang et al., 2020; Messner et al., 2021). We found that DIA was able to show alterations in the secretome of ADAM15 knockdown cells that other mass spectrometry-based methods were not able to detect. Among these altered proteins, DIA-MS identified a reduction in structural ECM components, such as type I collagen, and an increase in catabolic enzymes, including metallopeptidases and glycosidases upon ADAM15 knockdown. However, none of these proteins was a single-pass transmembrane protein and therefore a putative canonical substrate of ADAM15, so the mechanism(s) by which ADAM15 regulates them are unknown. Our study provides new information on the activity of ADAM15 and indicates that it can regulate cell surface and secretome levels of proteins involved in the regulation of cartilage homeostasis through an unknown post-transcriptional manner. This could potentially be through effects on intracellular trafficking or degradation, and implies that ADAM15 interacts with these proteins or their regulators during intracellular maturation and/or transport. Further detailed mechanistic studies are required to investigate this novel possibility, and to investigate whether other ADAMs have similar hitherto unexplored activities.

## Data Availability

The mass spectrometry proteomics data used in this study have been deposited to the ProteomeXchange Consortium via the PRIDE partner repository with the dataset identifier PXD042358 and PXD042402.

## Abbreviations

ADAM, a disintegrin and metalloproteinase; ANC, acetonitrile; DBCO, dibenzocyclooctyne; LC-MS/MS, liquid chromatography, tandem mass spectrometry; ManNAz, tetraacetylated N-Azidoacetyl-D-Mannosamine; OA, osteoarthritis; PDCD1LG2, programmed cell death 1 ligand 2; SLC26A2, sulfate transporter; SUSPECS, surface-spanning protein enrichment with click sugars; VASN, vasorin.
