# Supplementary figures and images for: Identification of membrane proteins regulated by ADAM15 by SUSPECS proteomics
Source: Front Mol Biosci. 2023 Jun 14;10:1162504. doi: 10.3389/fmolb.2023.1162504 (PMC10304831; doi:10.3389/fmolb.2023.1162504)

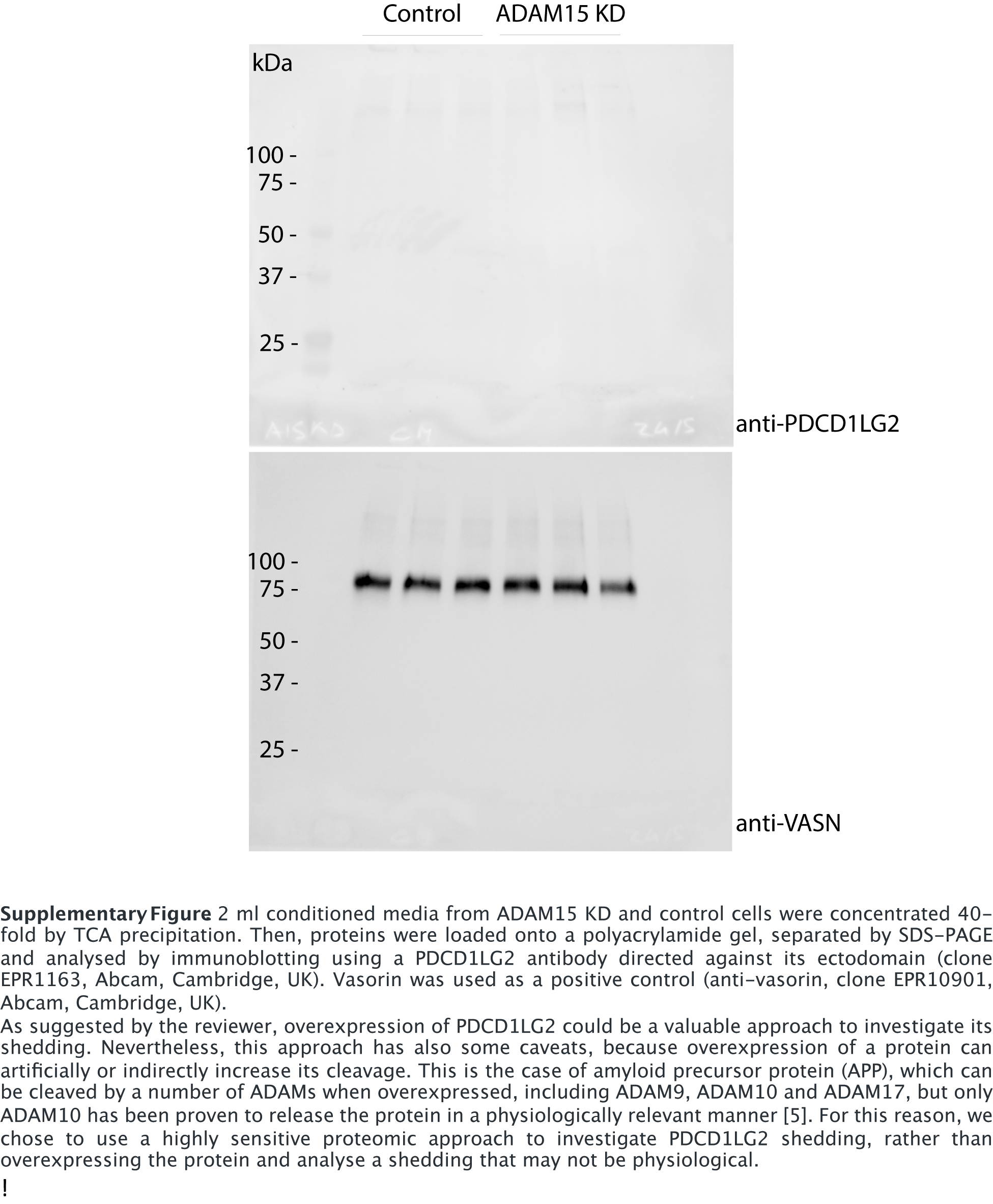

Supplement: Supplementary file 3 [file Image1.TIF]
